# Scoping Review of Dance for Adults With Fibromyalgia: What Do We Know About It?

**DOI:** 10.2196/10033

**Published:** 2018-05-10

**Authors:** Julia Bidonde, Catherine Boden, Soo Kim, Angela J Busch, Suelen M Goes, Emily Knight

**Affiliations:** ^1^ Division for Health Services Norwegian Institute of Public Health Oslo Norway; ^2^ School of Rehabilitation Science College of Medicine University of Saskatchewan Saskatoon, SK Canada; ^3^ Leslie and Irene Dubé Health Sciences Library University of Saskatchewan. Saskatoon, SK Canada; ^4^ School of Health Science Western University London, ON Canada

**Keywords:** fibromyalgia, exercise, dancing, scoping review, adult

## Abstract

**Background:**

Fibromyalgia is a chronic disorder characterized by widespread muscular tenderness, pain, fatigue, and cognitive difficulties. Nonpharmacological treatment options, such as physical activity, are important for people with fibromyalgia. There are strong recommendations to support engagement in physical activity for symptom management among adults with fibromyalgia. Dance is a mode of physical activity that may allow individuals with fibromyalgia to improve their physical function, health, and well-being. Dance has the potential to promote improved pain processing while simultaneously providing the health and social benefits of engaging in physical activity that contributes to symptom management and overall function rehabilitation. However, we are unaware of current evidence on dance as a nonpharmacological/physical activity intervention for adults with fibromyalgia.

**Objective:**

The aims of this study were to understand how dance is used therapeutically by individuals with fibromyalgia; to examine the extent, range and nature of research activity in the area; and to determine the value of undertaking a systematic review of interventions.

**Methods:**

We used and adapted the Arksey and O’Malley scoping framework. The search strategy involved a comprehensive search of main health and electronic social databases, trial registries and grey literature without language limits. Pairs of reviewers independently screened and extracted data and evaluated the methodological quality of randomized control trials.

**Results:**

Twenty-one unique records for 13 studies met inclusion criteria; the studies included mostly middle-aged women. Types of dance included were aerobic dance, belly dance, dance movement therapy, biodanza and Zumba. Intervention parameters were different among studies. Frequency varied between one to three times a week; all were done in small group settings. Studies evaluated a variety of outcomes in the symptoms, wellness, psychosocial, physical functioning, balance and fitness categories; no studies evaluated the safety or adverse events systematically which is a major weakness of the literature.

**Conclusions:**

There are few studies in the field of dance and fibromyalgia, suggesting research is in its infancy but slowly growing. They are of European and South American origin, focusing on female participants and a limited number of dance modes. Because the body of literature is small, of low quality and highly heterogeneous, we concluded that a systematic review of interventions on dance is not warranted at this time.

## Introduction

### Background

Fibromyalgia is a chronic disorder characterized by widespread muscular tenderness, pain, fatigue and cognitive difficulties [1,2]. The diagnosis is often complex, requiring a history of typical symptoms over time and the exclusion of a somatic disease by medical examination [1]. In addition to pain, fatigue, and cognitive difficulties, individuals with fibromyalgia may experience sleep and mood disturbances, anxiety, depression, difficulty with attention and concentration, as well as a range of gastrointestinal (eg, irritable bowel syndrome) and somatosensory (eg, hyperalgesia, allodynia, paresthesia) symptoms [1]. Symptoms of fibromyalgia can affect an individual’s quality of life, often negatively impacting family dynamics, productivity at work, and independence [2].

Fibromyalgia is common worldwide with the prevalence reported to be 2%-4% of the general population, and diagnosis in females outnumbering diagnosis in males [1,3]. Insights gained from research in the past several decades implicate numerous factors in its pathophysiology including changes in brain and neural structure and function, muscular physiology, hormonal factors, inflammatory markers, and genetic influences [4,5]. Individuals with fibromyalgia often experience comorbid illnesses, including musculoskeletal conditions, cardiovascular or endocrinological disorders, spondylosis/intervertebral disc disorders, interstitial cystitis bladder syndrome, chronic pelvic pain, temporomandibular joint disorder, and psychiatric disorders [6].

### Physical Activity and Dance

A substantial evidence base supports the use of physical activity for individuals with fibromyalgia. The latest European League Against Rheumatism guideline stated there is a strong recommendation to support both aerobic and resistance training in symptom management for individuals with fibromyalgia [7]. Physical activity is defined as any bodily movement produced by skeletal muscles resulting in energy expenditure [8]. Dance, a genre of physical activity, can be a social experience, an artistic expression, and a leisure activity, as well as a rigorous stimulus for physical fitness. We operationalize dance as a purposeful, deliberate, and expressive motion of the body caused by contraction of the skeletal muscles [9]. Dance may include music; and although dance movements could be called “functional” (eg, bending, walking, and reaching), the goal of dance is the deliberate and purposeful expression of the body itself through movement [10].

Benefits of dance for chronic conditions can be found in the literature; for example, increased functional and cardiovascular gains, motivation for participation [11], and quality of life [12], as well as a reduction in cardiovascular mortality [13], when compared to traditional exercise training. Emotional benefits were seen after dance-based exercise participation among older individuals [14]. One specific dance-based approach common in the literature is dance movement therapy (DMT), which has been defined as the psychotherapeutic use of movement that furthers the emotional, social, cognitive, and physical integration of the individual [15].This form of dance may include a variety of movement methods that have a systematic treatment approach and are goal-oriented [16]. DMT has been used for conditions including cancer [16], schizophrenia [17], depression [18], dementia [19,20], and Parkinson’s disease [21]. At the start of this scoping review, we were aware of two publications that include adults with fibromyalgia [22-24].

Dance contributes to the physical training of balance, coordination, strength, flexibility, aerobic capacity, bone health, and proprioception. Additionally, dance promotes increased motivation to exercise [25], attention and cognitive capacity [26], vitality [27], and positive effects on mood [28], everyday competencies, and social life [29]. Dance can also offer auditory, visual and sensory stimulation, motor learning, emotional perception, expression, and interaction. All these features make dance an “enriched environment” which stimulates the brain’s plasticity [29] and suggest that dance may be worth evaluating as a component of fibromyalgia management.

### Pain Processing and Social Bonding

Widespread pain and fatigue are hallmark symptoms of fibromyalgia and are known factors limiting an individual’s participation in treatment [30]. During physical activity, the muscular and physiological stress on the body stimulates the release of endorphins, which contributes to the sensation of an “activity high” and, potentially, a “social high” [31]. Evidence supports that both physical pain (the unpleasant experience that is associated with actual or potential damage to tissue) and social pain (the unpleasant experience that is associated with actual or potential damage to one’s sense of social connection or value) are processed with shared neural circuitry [32]. This supports the hypothesis that experiences in social and physical pain may be similar for the individual, such that individuals experiencing chronic physical pain are more likely to avoid activities for fear of inducing both social and physical pain [32,33]. Therefore, a social activity intervention may lead to improved treatment outcomes for adults with fibromyalgia by improving pain processing.

Dance is an engaging and enjoyable form of physical activity. Group or social dance facilitates social bonds, through working in synchrony (performing the same movements at the same time) [31,34]. Synchronization and physical exertion, such as through dance, independently elevate the pain threshold [31]. Moreover, dance can increase self-control, which impacts psychological health and therefore the experience of chronic pain [35]. Therefore, dance has the potential to promote improved pain processing while simultaneously providing the health and social benefits of engaging in physical activity that may contribute to symptom management for adults with fibromyalgia.

This scoping review aimed to: comprehensively examine and map the evidence related to dance in adults (ie, 18 years or older) with diagnosed fibromyalgia; to examine the extent, range and nature of research activity in the area; and to determine the value of undertaking a systematic review of interventions. Definitions used in this review are found in the glossary (Multimedia Appendix 1).

## Methods

Scoping review methodology is particularly useful for examining the breadth of the research in a specific topic area. We used and adapted the Arksey and O’Malley scoping framework [36]; adaptations (including a seventh step, knowledge dissemination, not reported in this manuscript) were driven by an intention to develop a feasible approach for reviewing the body of literature. The steps included identifying the research questions and relevant studies; selecting the studies and charting the data; collating, summarising and reporting the results; and ongoing consultation. A detailed description of these steps is outlined in our protocol [37].

The population, intervention, comparator, and outcome (PICO) criteria and the search strategy are presented in the Multimedia Appendices 2 and 3 and also in our protocol [37]. Pairs of reviewers independently screened citations for inclusion, extracted data and evaluated the methodological quality of randomized control trials (RCTs) using the Cochrane Collaboration Risk of Bias Tool [38]. Conflicts were resolved by consensus and with the aid of a third reviewer if needed. Criteria used for screening, extracting and methods for quality evaluation are provided in our protocol [37].

We used frequencies and percentages to describe nominal data. We shared the findings with the researchers and patients engaged with the Cochrane Fibromyalgia and Physical Activity team led by one of the authors (JB), and we integrated all responses into this review.

## Results

### Identifying and Selecting Relevant Studies

**Figure 1:**
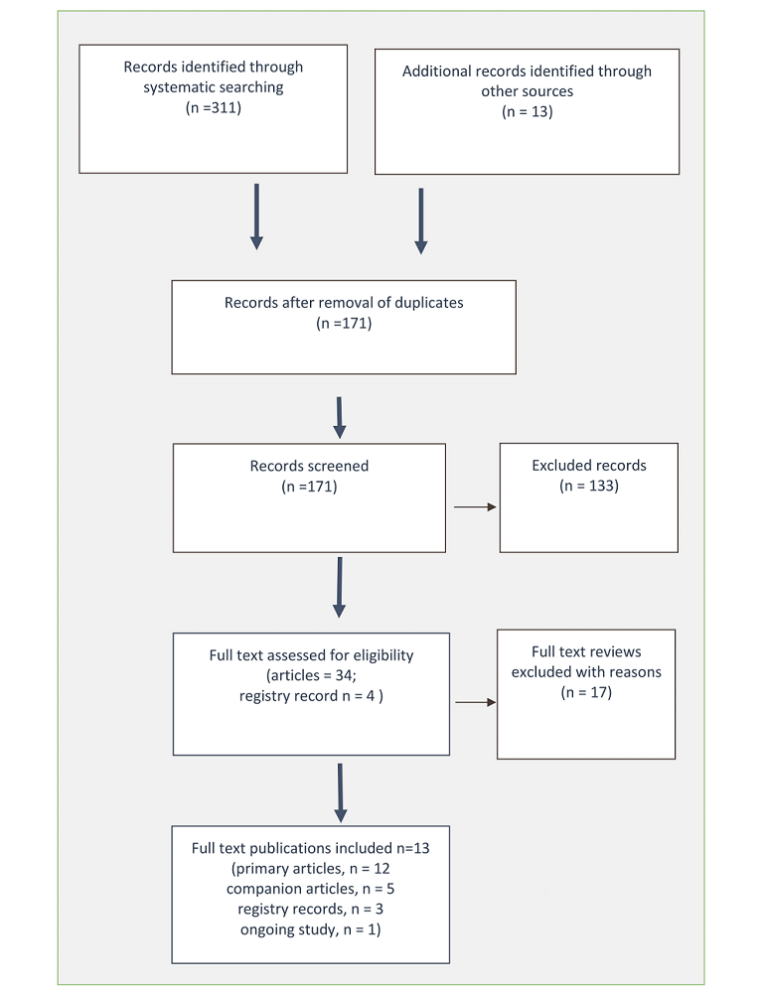
The Preferred Reporting Items for Systematic Reviews and Meta-Analyses (PRISMA) flow diagram.

The search of the databases, clinical trials registries, and citation tracking yielded 171 citations after duplicates were removed. Figure 1 presents results of the literature search and flow of articles. Search of fibromyalgia association websites did not yield research reports. In total, we screened 171 publications at title and abstract phase and excluded 133 not meeting the inclusion criteria. The full-text of 34 articles and four trial registry records were screened, and 21 records (ie, unique, companion, and trial registry records) for 13 studies were included [22-24,39-56]. Of the four trial registry records, three were protocols for full-text publications [40,47,54] and one was for a study currently recruiting [48]. Five studies each published two articles for the same study, and the second publication is considered a companion article for the same study: those are Assunçao Júnior [39,53] Bojner Horowitz [24,43], Carbonell-Baeza [46,56], Collado-Mateo [49,50], and Lopez Rodriguez [22,23]. Studies by Lopez Rodriguez were a pilot trial and follow up conducted consecutively; we believe these two publications have substantial overlap in their samples, and with a trial registry record [54]. A publication summary is presented in Table 1.

### Charting and Collating the Data

**Figure 2:**
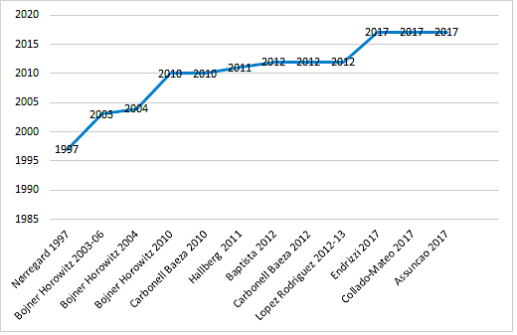
Dance and fibromyalgia publication's timeline.

Publications were original peer-reviewed journal articles; designs were uncontrolled before and after (n=4), controlled before and after (n=2), qualitative (n=2), and RCTs (n=6). All but one study [55], were published after 2003 (range 1997-2017; see Figure 2). Two publications were from South America and the remaining from Europe. Eleven articles were written in English, and one in Spanish. Dance interventions modes included aerobic dance, belly dance, biodanza, DMT, and Zumba. Most of these interventions included dance and another component (eg, DMT+theatre+cultural events).

Outcomes measured fell within seven domains: symptoms (ie, pain), wellness (eg, overall health), psychosocial (ie, self-image), physical function, balance, and fitness (ie, muscle strength), and other (ie, body composition). We have summarized outcomes and outcome measures in Multimedia Appendix 4. No studies assessed adverse events systematically, and narrative reports were included in few instances [41,46,50,52], generally relating to an acute increase in pain (see Multimedia Appendix 5). The number of withdrawals was reported in nine studies [22,39,41,44-46,50,51,55].

**Figure 3:**
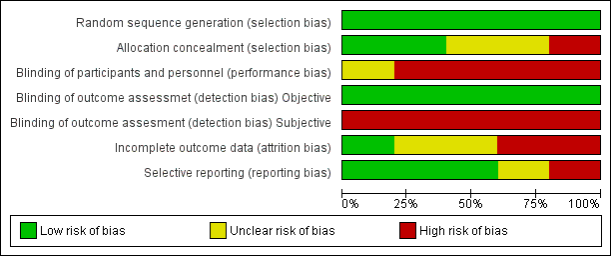
Methodological quality of randomized controlled trials.

**Figure 4:**
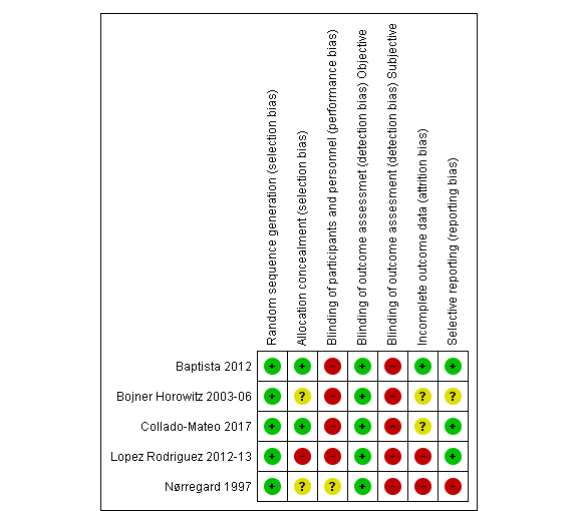
Methodological quality of randomized controlled trials.

We evaluated the methodological quality of five published RCTs [22,24,41,50,55] (Figures 3 and 4) to determine the value of undertaking a systematic review of interventions. Results demonstrated problems of selection, performance, detection and reporting biases. Other factors affecting study quality are assessment of a large number of outcomes, diversity on the psychometric and other outcome measures, and clinical heterogeneity.

### Participants

Participants were mainly middle-aged females with a fibromyalgia diagnosis (ACR 1990, n=11; ACR 2011, n=1; unclear criteria, n=1); one study recruited males but its final sample composition was unclear [55]. The total number of participants across studies was 488 (median 36). Participant age ranged between 30 and 68 years. Duration of fibromyalgia (years since diagnosis) varied from 2 to 35 years (not reported in three studies). Participants who were taken medications needed to be on a stable course of pharmacological treatment before starting the intervention, and have no contraindications for physical activity (eg, uncontrolled hypertension). The inclusion criteria of one study specified that participants needed to meet the criteria for depression [51]; two studies specified inclusion criteria for pain levels to be between 3-8 on a visual analog scale [23,51].

### Intervention and Music

Interventions were performed once (n=5), twice (n=4) or three times a week (n=2). Intensity was not specified in five studies, set as a low intensity (n=2), worded as listening to their bodies or not exceeding pain thresholds (n=2) or set at 40%-50% of oxygen consumption (VO_2_; n=1). Intervention duration ranged between 50 to 120 minutes and length between 8 to 24 weeks; only two studies reported long-term follow up times (see Table 2). There was limited information about the qualifications of the instructor or the setting (group or individual). Five studies lack information on the instructor’s qualifications [22-24,43,46,48,55], two mentioned a physiotherapist with experience in dance [41,45], one a student with training in dancing [39,53], one professional kinesiologist and dance teacher [49,50] and the rest made reference to “study leader.”

**Table 1:** Publication summary.

Characteristics		Value
**Year of Publication, n (%)**		
	1997	1 (8)
	2003-10	4 (31)
	2011-17	7 (54)
	Ongoing	1 (8)
**Continent, n (%)**		
	South America	2 (15)
	Europe	11 (85)
**Language, n (%)**		
	English	12 (92)
	Spanish	1 (8)
**Design, n (%)**		
	Randomized control trial	6 (46)
	Controlled before and after	2 (15)
	Uncontrolled before and after	4 (31)
	Grounded theory	1 (8)
**Mode, n (%)**		
	Aerobic dance	1 (8)
	Belly dance	1 (8)
	Biodanza	3 (23)
	Dance movement therapy	5 (38)
	Zumba	2 (15)
	Activity, recovery and balance	1 (8)
**Type of publication, n**		
	Primary article	12
	Companion article (published protocol or additional publication)	5
	Ongoing (ie, trial registry record status recruiting)	1

We found limited information on how music was used (see Multimedia Appendix 6), such as to inspire spontaneous movement, creativity and emotional expression [22,23,44-46]. In some studies, music also involved a receptive listening experience with the aim of facilitating dialogue, where the participants engaged in a relational process with peers during the sessions [22,23,45,46].

In Hallberg [52] the women described dance as an enjoyable and desirable activity “…*I’ll pretty much dance to every song during a dance evening…it was so much fun*.” Although dance continued to be a valued activity in participants’ lives, their narratives closely interlaced the physical effort it represented, the persistence of pain, and limitations it caused. However, the sense of joy and perseverance prevailed: *“But it’s worth it, you have to live.”* Madeiros [53] followed up after a 3-month Zumba intervention, and the women reported benefits on sleep quality, pain, self-esteem, and physical functioning.

### Gaps in the Literature

We were unable to conduct comparative analyses of key concepts across studies due to lack of consistency in conceptual definitions of dance. Participants’ medications were not described in all studies, thus could not be summarized. In addition to lack of systematically measuring adverse events, no studies addressed concepts of communication (eg, isolation), challenges or barriers to participation or implementation, acceptability, feasibility or applicability for clinical practice. There was no information available on cost or equipment.

**Table 2:** Intervention characteristics.

Characteristics^a^		Value, n (%)
**Times per week**		
	1x/week	5 (45)
	2x/week	4 (36)
	3x/week	2 (18)
**Intensity**		
	Respect their body rhythm and limits	1 (9)
	Ability to change intensity difficulty	1 (9)
	40%-50% VO_2_ max^b^	1 (9)
	Low	2 (18)
	Not exceeding pain	1 (9)
	No reported	5 (45)
**Duration**		
	50 to 60 minutes	7 (64)
	61 to 120 minutes	3 (27)
	Not reported	1 (9)
**Length of intervention**		
	8 to 11 weeks	3 (27)
	12 to 16 weeks	7 (64)
	>16 weeks	1 (9)
	Follow up (ie, after end of intervention)	2 (18)
**Delivery mode**		
	Individual	1 (95)
	Small group or group	4 (36)
	Individual, pair and group	2 (18)
	Not reported	4 (36)

^a^Qualitative study and ongoing not included.

^b^VO_2_: maximal oxygen uptake.

## Discussion

### Principal Findings

There has been some interest in dance as a potential nonpharmacological intervention in fibromyalgia, yet the body of knowledge remains small. Most studies were published after 2003, included middle-aged women, and used a small number of dance modes (belly dance, DMT, aerobic dance, biodanza, Zumba). Currently, there is a broad variation across studies (ie, design, mode, delivery mode, intervention parameters); the creation and agreement of consistent terminology, starting with the definition of dance, would be beneficial.

Dance was used in the studies as a form of exercise training (eg, aquatic biodanza), or performed because of its artistic or creative nature (eg, DMT). Dance was one of a multi-component (or mixed) intervention; this is an important consideration for practitioners and individuals wishing to engage in dance. Interaction with others was important; dance was conducted in groups or small groups to help socialization. This is not surprising as socialization holds potential to affect pain processing [32] thereby potentially improving treatment outcomes for individuals with fibromyalgia [37]. Music was used as a tool for creativity and expression, as well as socialization but its use was not well defined. Researchers need to provide better descriptions concerning parameters of the intervention, such as exercise frequency, intensity, time (duration), type (and mode), use of music, and instructor qualifications.

Dance mode, outcomes, and outcome measures were heterogeneous, which poses challenges for synthesizing evidence. Additionally, the risk of bias assessment of RCTs showed a high risk of selection bias related to subject allocation, performance and detection bias related to blinding and reporting biases. None of the included studies evaluated safety or adverse events systematically, which is a major weakness of these studies, and consistent with the fibromyalgia and exercise literature more generally [57]. The lack of data on acceptability, feasibility, applicability, and cost-effectiveness represent drawbacks to informing clinical practice.

Future research may wish to consider the individual effects of socialization, music, and physical effects of dance itself to better understand the role of dance in enhancing treatment outcomes among individuals with fibromyalgia.

Some limitations to this scoping review exist. First, we have focused on providing the breadth rather than depth of information on this topic, so questions remain regarding the effectiveness of this intervention. Second, no electronic database contains all the information needed for a research project; the search is limited to what was available to researchers. Finally, we are aware others might define dance in a more or less inclusive way than we have done, consequently capturing somewhat different literature for review.

Acknowledging that these studies represent initial steps in the field, prudence is necessary when recommending dance to individuals with fibromyalgia as we do not yet have a proper understanding of its benefits and harms. This lack of evidence will negatively impact knowledge translation efforts, such as safely integrating dance into clinical management approaches.

### Conclusion

This scoping review is, to our knowledge, the first systematic and rigorous synthesis conducted of studies reporting on dance as a nonpharmacological intervention for adults with fibromyalgia. The study demonstrates there is a small body of evidence using interventions such as belly dance, DMT, aerobic dance, biodanza, and Zumba mostly conducted in middle-aged women. Safety issues were not assessed systematically or reported, representing a major gap in the current literature. Lack of common intervention approaches and outcome measures as well as standardization in reporting outcomes presents a barrier to pooling data. To date, adults with fibromyalgia interested in engaging in dance programs for control of symptoms have little evidence to aid in their decision-making. As this body of research grows, our understanding of dance in adults with fibromyalgia will improve and provide meaningful information about the potential role of dance in symptom management, physical and mental health of adults with fibromyalgia and for health practitioners working with these individuals.

## Appendix

Multimedia Appendix 1 Glossary of terms.

Multimedia Appendix 2 PICO criteria.

Multimedia Appendix 3 Search strategy.

Multimedia Appendix 4 Outcome and outcome measures (ie, scale or technique).

Multimedia Appendix 5 Adverse events.

Multimedia Appendix 6 Dance intervention classification and use of music.

## Abbreviations

DMT: dance movement therapy
PICO: population, intervention, comparator, and outcome
PRISMA: Preferred Reporting Items for Systematic Reviews and Meta-Analyses
RCT: randomized control trial
VO_2_ max: maximal rate at which an individual can process oxygen
