# Supplementary material for: Scoping Review of Dance for Adults With Fibromyalgia: What Do We Know About It?
Source: JMIR Rehabil Assist Technol. 2018 May 10;5(1):e10033. doi: 10.2196/10033 (PMC5968214; doi:10.2196/10033)
Supplement: Multimedia Appendix 1 [file rehab_v5i1e10033_app1.pdf]

Glossary of Terms

Term	Definition
Aerobic dance	Activities combining dancing and other continuous vigorous activity required to produce a training effect on the heart and lungs accompanied by lively music; the movements are simple but planned. Movements include a combination of upper and lower body and come from a variety of popular sources including tap, jazz, etc. This type of class is a viable method for gaining cardiorespiratory fitness and was popular in the 1980s.
Ballroom dancing	Social dance usually performed by couples in dance halls or at social gatherings. These dances became popular in the 1920s and 1930s and include dances like the waltz, tango, Charleston, salsa, rumba, cha-cha-cha and the quickstep.
Bias	A systematic error that is introduced into sampling or testing by selecting or encouraging one outcome or answer over others. [1]
Biodanza	Biodanza is a process of global human development; it is an invitation to get into movement, to express and develop oneself among a group of people. Biodanza follows a precise combination of exercises, dances and music. It offers the possibility to progressively rehabilitate one's life force, one's « joie de vivre », and promotes a general sensation of well-being
Belly dancing	A dance originally from Western Asia in which a woman moves primarily torso and hips in undulating movements.
Zumba	An aerobic fitness program featuring movements inspired by various styles of Latin American dance and performed primarily with a choreographed dance to Latin American dance music.
Scoping review	Preliminary assessment of potential size and scope of available research literature. Aims to identify nature and extent of research evidence (usually including ongoing research).[2]
Ongoing trial	A trial (registered in an international database) actively assigning humans or groups of humans to one or more health related intervention to evaluate the effect

	on health outcomes
Randomized controlled trial	A study in which people are allocated at random to receive one of several clinical interventions. One of these interventions is the standard of comparison or control. The control may be a standard practice, placebo or no intervention. In addition to the randomization principle, the trials are often carried out with blinding such that neither the participant nor the outcome assessor may be aware of group allocation.

1. Merriam-Webster Dictionaries. Bias Springfield, MA: Merriam-Webster Inc.; 2018 [cited 2018 April 7]; Available from: https://www.merriam-webster.com/dictionary/bias.
2. Colquhoun HL, Levac D, O'Brien KK, Straus S, Tricco AC, Perrier L, et al. Scoping reviews: time for clarity in definition, methods, and reporting. J Clin Epidemiol. 2014;67(12):1291-4. doi: 10.1016/j.jclinepi.2014.03.013.
