# Supplementary figures and images for: Scoping Review of Dance for Adults With Fibromyalgia: What Do We Know About It?
Source: JMIR Rehabil Assist Technol. 2018 May 10;5(1):e10033. doi: 10.2196/10033 (PMC5968214; doi:10.2196/10033)

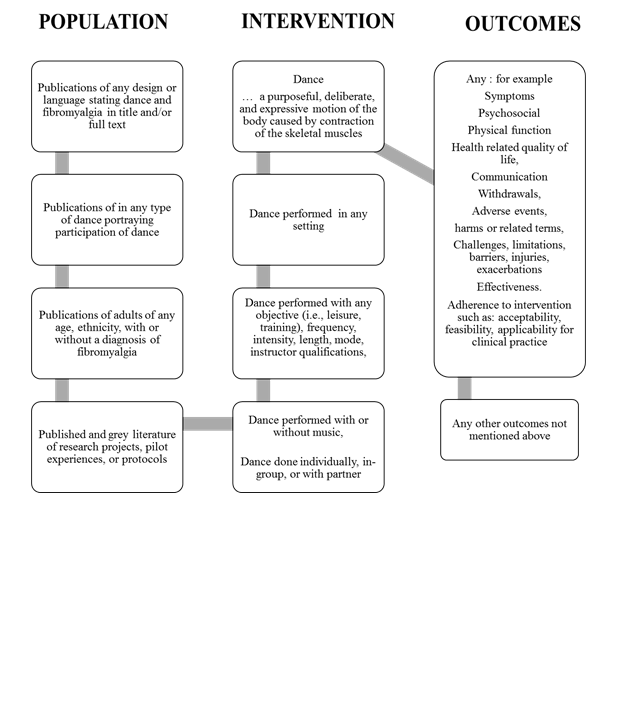

Supplement: Multimedia Appendix 2 [file rehab_v5i1e10033_app2.png]
