# Supplementary material for: Scoping Review of Dance for Adults With Fibromyalgia: What Do We Know About It?
Source: JMIR Rehabil Assist Technol. 2018 May 10;5(1):e10033. doi: 10.2196/10033 (PMC5968214; doi:10.2196/10033)
Supplement: Multimedia Appendix 3 [file rehab_v5i1e10033_app3.pdf]

Databases:

- Medline in-process and other nonindexed citations (Ovid)—1946 to January 5, 2018
- Embase and Embase Classic (Ovid)—1947 to January 5, 2018
- Cochrane Library (Wiley) – inception to January 5, 2018
- PsycINFO (Ovid)—1806 to January 5, 2018
- Cumulative Index of Nursing and Allied Health Literature (CINAHL) (EBSCO)—1937 to January 5, 2018
- Literature in the Health Sciences in Latin America and the Caribbean (Literatura Latino Americana em Ciências da Saúde, LILACS) – inception to January 5, 2018
- Allied and Complementary Medicine (AMED) (Ovid)—1985 to January 5, 2018
- International Bibliography of Theatre and Dance (EBSCO)—1984 to January 5, 2018
- Physiotherapy Evidence Database (PEDro) – inception to January 5, 2018
- TRIP – inception to January 5, 2018
- ProQuest Theses and Dissertations—1997 to January 5, 2018
- Web of Science Core Collection (Thomson Reuters)—1900 to January 5, 2018
- World Health Organization International Trial Registry Portal – inception to January 5, 2018

- ClinicalTrials.gov – inception to January 5, 2018

Other resources:

- We searched the bibliographies of relevant studies and reviews.

- Corresponding authors of previously found dance randomized controlled trials will be contacted regarding their knowledge of ongoing studies or groups involved in the area.

- An a priori set of fibromyalgia associations will be selected and their associations' webpages will be screened for annual reports or findings that these associations produce based on their own research, which will be retrieved

Medline Search Strategy

1 Dancing/

2 Dance Therapy/

3 danc*.tw.

4 (movement adj5 therap*).tw.

5 (biodanza or biodance).tw.

6 ((Body or artistic or synchroniz* or music* or rhythm* or express*) adj movement*).mp.

7 (rhythm* adj activit*).mp.

8 zumba.mp.

9 choreograph*.mp.

10 1 or 2 or 3 or 4 or 5 or 6 or 7 or 8 or 9

11 Fibromyalgia/

12 fibromyalgi$.tw.

13	fibrositis.tw.
14	fibromialgia.tw.
15	11 or 12 or 13 or 14
16	10 and 15
