# Supplementary material for: Scoping Review of Dance for Adults With Fibromyalgia: What Do We Know About It?
Source: JMIR Rehabil Assist Technol. 2018 May 10;5(1):e10033. doi: 10.2196/10033 (PMC5968214; doi:10.2196/10033)
Supplement: Multimedia Appendix 4 [file rehab_v5i1e10033_app4.pdf]

**Domain- Outcome**	**Scale - technique**
Symptom	
Pain Intensity/Interference	Visual Analog Scale (VAS)
	SF36 bodily pain
	FIQ pain, and interference in daily activity
	McGill-Melzack questionnaire
	EQ-5D-5L pain/discomfort
Depression	Beck Depression Inventory (BDI)
	FIQ depression
	Montgomery Åsberg Depression Rating Scale
	Center for Epidemiologic Studies Depression Scale (CES-D)
Anxiety	State Anxiety Inventory (STAI)
	FIQ anxiety
Depression & Anxiety	Comprehensive psychopathologic rating scale
	Hospital Anxiety and Depression Scale (HADS)
	EQ-5D-5L anxiety & depression
Fatigue or Vitality or Tiredness	SF36 vitality
	FIQ Fatigue and morning tiredness
	Epworth Sleepiness Scale
Stiffness	FIQ Stiffness
Tender Points	18 tender points
Sleep	FIQ Sleep
	Pittsburgh Sleep Quality Index (PSQI)
Wellness	
Quality of Life	FIQ total
	EuroQol-5
Overall Health	SF36 General Health
	Global assessment of well-being and pain
Psycho-social	
Self-image	Body disorder Dysmorphic Examination
Social Support	SF36 role social
	SF 36 emotional supports
Mental Health	SF36 mental health
	Sense of Coherence
Coping	Vanderbilt Pain Management Inventory (VPMI)
	Video Interpretation(mobility, life energy, and movement pain)
Self - Perception	Self-figure drawing
Personality	Swedish Universities Scale of Personality
Treatment Satisfaction	1 to 5/6 Likert scale
	Ranking of dance, music or drawing (1-3 scale)
Self Esteem	Rosenberg Self-Esteem Scale (RSES)
Self Efficacy	General Self-Efficacy Scale
Fear of Falling	VAS (no fear to extreme fear)
Physical Function	
Functioning	SF36 physical function,
	SF36 limited capacity to work,
	Functional Senior Fitness Test Battery

	FIQ physical function
	EQ-5D-5L Mobility, usual activities & self-care
Balance	
Dynamic and static balance	Mini Balance Evaluation System Test
	8 feet up and go test
	Functional reach
	Blind Flamingo Test
	Clinical Test of Sensory Integration of Balance
Fitness	
Cardio-Sub Max	6 Minute Walk Test
Strength	Jamar dynamometer (quadriceps)
	30-second chair stand test
	Handgrip strength by digital dynamometer
Flexibility	Chair sit and reach test
	Back scratch test
Other	
Biological	Blood, cortisol, prolactin, neuropeptide Y, Dehydroepiandrosteronsulphate,
Therapeutic Process	
Patient participation	The Therapist Experiential Session Form
Body composition	1 to 5 Likert Scale
	Body Mass Index
	Body fat percentage
	Muscle mass
	Waist circumference
