# Supplementary material for: Scoping Review of Dance for Adults With Fibromyalgia: What Do We Know About It?
Source: JMIR Rehabil Assist Technol. 2018 May 10;5(1):e10033. doi: 10.2196/10033 (PMC5968214; doi:10.2196/10033)
Supplement: Multimedia Appendix 5 [file rehab_v5i1e10033_app5.pdf]

Author	Intervention	Adverse Events
Assuncao 2017	Zumba	none mentioned
Baptista 2012	Belly Dance	“The patients in the dance group initially reported an increase in pain and fatigue in the first four weeks, which decreased over time.”
Bojner Horowitz 2003-06	DMT	none mentioned
Bojner Horowitz 2004	DMT	none mentioned
Bojner Horowitz 2010	DMT	none mentioned
Carbonell Baeza 2010	Biodanza	“During the study period, no participant reported an exacerbation of FM symptoms beyond normal flares, and there were no serious adverse events”
Carbonell Baeza 2012	Biodanza	none mentioned
Collado Mateo 2017	Zumba/exerg	"No adverse effects were noted"
Endrizzi 2017	DMT	none mentioned
Hallgerg 2011	Activity, recovery and balance	“I’ll pretty much dance to every song during a dance evening. Then we go home after the last dance and getting out of the car can be sheer hell, my feet ache, my legs ache, my back aches and the day after you can't really do a whole lot.”
		“I had to call the doctor after I had been to a wedding and tell him that I'm going to need a couple of shots. I had been dancing and had a blast but I know that I'll end up having a lot of pain if I dance an entire evening”
Lopez Rodriguez 2012-13	Biodanza	none mentioned
Nørregaard 1997	Aerobic Dance	none mentioned
NCT02144116 /ongoing	DMT	Recruiting
