# Supplementary material for: Scoping Review of Dance for Adults With Fibromyalgia: What Do We Know About It?
Source: JMIR Rehabil Assist Technol. 2018 May 10;5(1):e10033. doi: 10.2196/10033 (PMC5968214; doi:10.2196/10033)
Supplement: Multimedia Appendix 6 [file rehab_v5i1e10033_app6.pdf]

Author/ year	Dance Intervention
Assunçao Júnior & Araujo 2017	Zumba
	**Music:** "People who practice Zumba get involved with the rhythms, the movements, and choreographies, all of which are easy to learn. There is no idea of right or wrong; each individual dance at his or her own pace and rhythm, getting carried away by the sound of the music itself."
Baptista 2012	Belly Dance - Supervised sessions and home program
	**Music:** "The participants received a compact disc with music"
Bojner Horowitz 2003-06	Dance Movement Therapy
	**Music** "was used in the sessions and at each treatment session, patients voted on desired music genres, with the majority determining the decision. Five different genres of music were presented: pop, instrumental, classical, folk music, and rhythmic world music. The patients chose rhythmic world music for use in 17 sessions. Three of the sessions used classical music".
Bojner Horowitz 2004	Dance Movement Therapy + video self-interpretation
	**Music:** "Patients were invited to move spontaneously to music focusing on one body part at a time: feet, knees, hips, shoulders, elbows, wrists, and head"
Bojner Horowitz 2010	Dance Movement Therapy + Theatre + Cultural Activities
	**Music:** "The patients visited the following cultural activities during the follow-up period …: Theatre performances, dance performances, music concerts…"
Collado Mateo 2017	Exergame; Zumba + postural control + coordination
	**Music**: No information
Carbonell Baeza 2010	Biodanza (verbal phase+living experience)
	**Music: "…**which involves moving/dancing according both to the

	suggestion given by the facilitator and the music played**.** The movements should express the emotions elicited by the songs (*12) as well as be a response to other peers' presence, proximity, and feedback."
Carbonell Baeza 2012	Biodanza (verbal phase+living experience)
	**Music: "…**given by the facilitator and the music played. The movements should express the emotions elicited by the songs (~12) as well as be a response to other peers' presence, proximity and feedback."
Endrizzi 2017	Dance Movement Therapy
	**Music:** no information
Hallberg 2011	"Tapes or CDs with relaxing sounds, for example lap of waves, twitter of birds and calm music that one appreciates can sometimes also lead to absence of pain."<br>"My energy level is at its highest when I'm doing something fun, when I go out dancing, which I love doing. People are amazed that I can handle dancing. But it makes me feel better. I think that what happens is that body muscles relax with the music. You kind of relax from the music, it's a little psychological, you confuse the pain."
Lopez Rodriguez 2012-13	Aquatic Biodanza (minor description)
	**Music: "…** different types of basic movements of expression and creative dance were carried out, including upper and lower members, in accordance with the music, expressing emotions aroused by the melodies, by the presence of others and by the interaction between them…"
Nørregard 1997	Aerobic Dance
	**Music:** Not mentioned
NCT02144116	Dance Movement Therapy

(ongoing)
